# Sequential Percutaneous Approach for Severe Mitral and Aortic Regurgitation

**DOI:** 10.7759/cureus.6619

**Published:** 2020-01-10

**Authors:** Claudio Guerreiro, Ana Raquel Barbosa, João Almeida, Bruno Melica, Pedro Braga

**Affiliations:** 1 Cardiology, Centro Hospitalar de Vila Nova de Gaia, Vila Nova de Gaia, PRT

**Keywords:** mitral valve regurgitation, aortic valve regurgitation, heart failure, transcatheter aortic valve implantation, percutaneous mitral valve repair.

## Abstract

Percutaneous approach for valvular heart disease is accepted as a safe and feasible strategy to treat patients considered at high surgical risk with aortic stenosis and mitral regurgitation. Additionally, the growing experience led to the increasing use of transcatheter aortic valve implantation in several indications, such as in pure aortic regurgitation. The authors present the case of a 72-year-old woman with prohibitive surgical risk due to several comorbidities, including a chronic arterial dissection from the right carotid artery to the femoral arteries bilaterally that presented with signs and symptoms of heart failure, having a transthoracic echocardiography that showed severe functional mitral and aortic regurgitation. Combined percutaneous intervention for multivalvular disease in this clinical scenario is challenging, and the reported experience is still scarce and limited to inoperable patients.

## Introduction

Experience with percutaneous valvular intervention has been steadily increasing, allowing operators to use this technology in different clinical scenarios [1,2]. A few series report the feasibility of double (concomitant or sequential) aortic and mitral valve intervention, although the performance of both procedures in the same patient has only been sporadically proposed [3-6]. Also, although feasibility has been demonstrated, pure aortic regurgitation (AR) poses a challenge on transcatheter aortic valve implantation (TAVI) due to the absence of annular or leaflet calcifications required for secure anchoring of transcatheter heart valves, increasing the risk of dislocation [7]. Despite the fact that these patients have been excluded from clinical trials, when deemed at high or prohibitive surgical risk they may still benefit from a percutaneous approach [8].

## Case presentation

**Figure 1:**
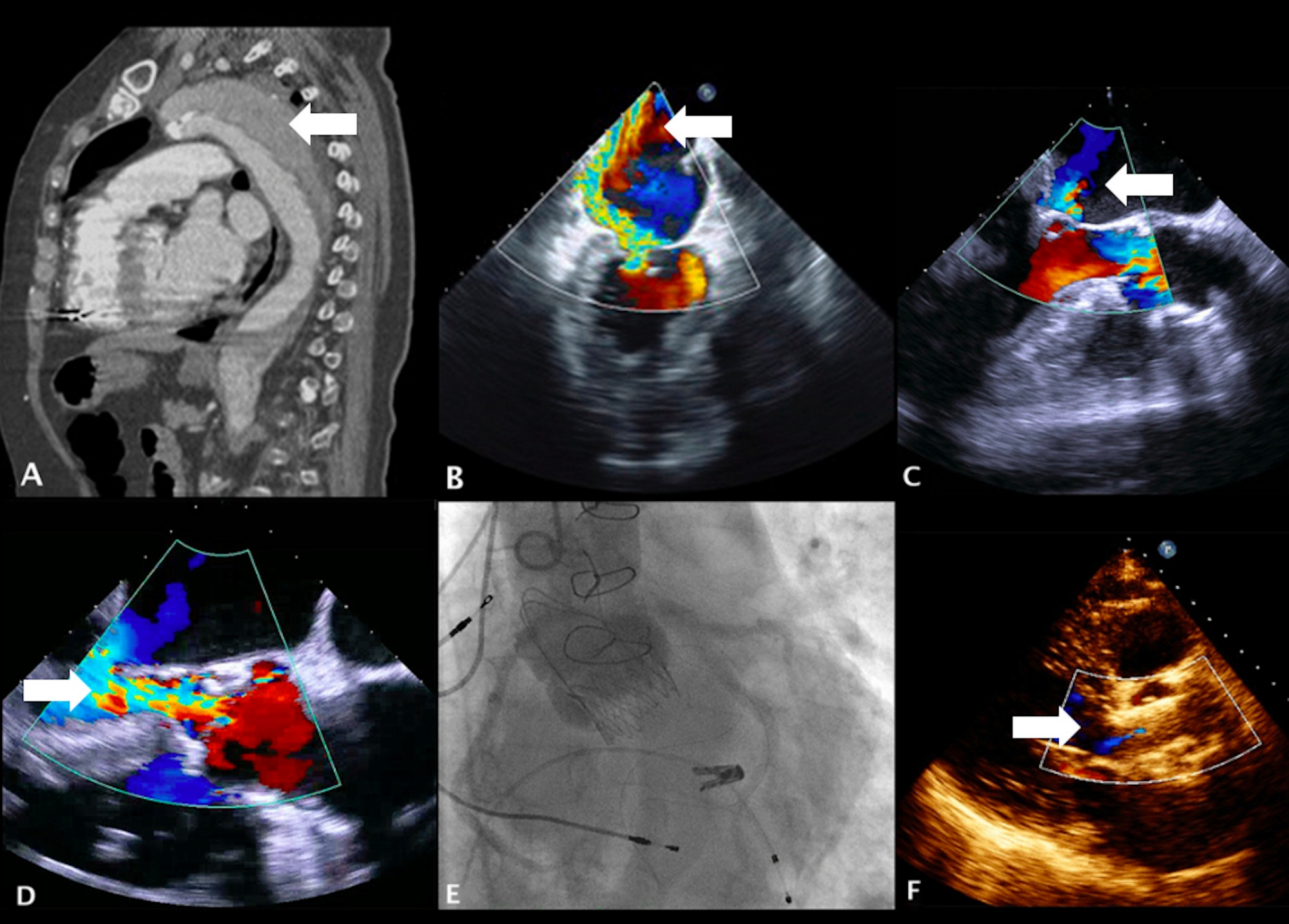
Sequential percutaneous approach for severe mitral and aortic valve regurgitation: pre- and post-procedural evaluation. (A) Sagittal reconstructed computed tomography image of the aorta, showing chronic dissection of thoracic aorta (white arrow). (B) Transesophageal echocardiography with eccentric severe mitral regurgitation reaching left atrial roof (white arrow). (C) Significant improvement of mitral regurgitation after percutaneous mitral valve repair (white arrow). (D) Severe aortic regurgitation before transapical aortic valve implantation (white arrow). (E) Final result of transcatheter aortic valve implantation, without stenosis, regurgitation or coronary obstruction (mean gradient 11 mmHg). (F) Mild aortic paravalvular leak in six-month follow-up transthoracic echocardiography (white arrow).

We report the case of a 72-year-old woman with arterial hypertension, stage IV chronic renal disease, history of idiopathic pulmonary embolism in 2012 and complete atrioventricular block in 2014, treated with implantation of a permanent bicameral pacemaker. She had undergone emergency ascending aorta reconstruction with a Dacron graft for a type A aortic dissection in 2002, persisting a chronic dissection from the right carotid artery to the femoral arteries (Figure 1A). One year prior to the admission, she developed symptoms of heart failure, complaining of progressive low-effort dyspnea, peripheral edema and bilateral lung congestion (New York Heart Association [NHYA] class III). The diagnostic workup showed a dilated left ventricle (indexed end-diastolic left ventricle volume 79 mL/m^2^) with moderate depression of systolic function (ejection fraction 40%), mitral annulus dilation (44 mm) and restriction of mitral valve posterior leaflet, contributing to severe functional mitral regurgitation (MR) (effective regurgitant orifice area 0.40 cm^2^, regurgitant volume 75 mL) (Figure 1B), and coexisted eccentric moderate AR (vena contracta 0.5 cm, color Doppler grade 3+/4+). Coronary angiography did not show obstructive coronary epicardial disease. Albeit optimal medical therapy, she remained highly symptomatic. After Heart Team evaluation, she was considered a high surgical risk candidate for valve replacement (logistic EuroSCORE 59.45%, STS score 5.35%, previous thoracic surgery). Considering the risk, the technical difficulties in the aortic approach and the degree of each valvular dysfunction, we decided first for percutaneous mitral valve repair with the MitraClip device system (Abbott Vascular, Menlo Park, CA) implanting one clip with success, without acute and 30-day adverse events and achieving an improvement to a color Doppler grade 2+/4+ MR, without significant increase in transmitral gradient (Figure 1C). After an initial clinical improvement, at six-month follow-up, the patient developed intolerance to daily activities and pulmonary congestion. Transesophageal echocardiography showed persistence of a good result of previous mitral intervention (color Doppler grade 2+/4+ MR) and a severe AR (vena contracta 0.6 cm, color Doppler grade 4+/4+; Figure 1D) with moderately depressed left ventricular (LV) ejection fraction (40%). The case was reevaluated in Heart Team, and it was decided to perform a transapical aortic valve implantation. A self-expandable, slightly oversized Symetis ACURATE TA^TM^ (Symetis Inc, Ecublens, Switzerland) bioprosthesis size M was implanted, with success and without signs of stenosis, prosthesis AR or coronary obstruction by the stent frame (Figure 1E). The patient was discharged five days after the procedure without major adverse cardiac and cerebrovascular events. At six-month follow-up, she remained in NYHA class II/IV, without admissions for decompensated heart failure, persisting a normal aortic valve implantation and function, with mild aortic paravalvular leak and mild-to-moderate MR (Figure 1F); LV function remained unchanged.

## Discussion

Concomitant aortic and mitral regurgitation are poorly tolerated by patients, because of the severe volume overload, with severe LV dilatation and loss of the protective mechanism of premature mitral valve closure, that would otherwise limit the amount of backward flow to the left atrium and pulmonary veins caused by AR. These patients have a high incidence of postoperative LV dysfunction and reduced postoperative survival [9].

It has been shown that high surgical risk patients benefit from valvular percutaneous approaches [1,2]. A few series report the feasibility of double aortic and mitral valve intervention, although the performance of both procedures in the same patient has only been sporadically proposed [3-6]. Kische et al. reported the experience of 254 patients referred for TAVI; 17 patients had pre-procedural severe MR and 12 of them were subsequently submitted to mitral valve repair with the MitraClip device due to exacerbation of heart failure symptoms. The procedural success was 100%, without major adverse events or 30-day mortality. There was a significant improvement of NYHA functional class (3.5±0.5 to 1.8±0.6; p<0.0001) and an overall increase in walking distance in six-minute walk tests (181.6±98.3 to 382±76.1 m; p<0.01). All patients remained with MR≤1+ and prosthetic aortic insufficiency ≤1+ [3].

Usually, aortic valve is the first to approach in order to reduce left ventricle overload and consequently the functional component of the MR. In this case, initially we assumed that approaching the latter would be safer and could improve the clinical status of our patient, considering also the off-label indication of TAVI for AR. TAVI with either balloon-expandable or self-expandable prosthesis are established therapeutic options for severe aortic stenosis in high surgical risk, and are being increasingly used in the intermediate profile as well [10]. Patients with symptomatic severe pure AR have poor prognosis and should be offered surgical aortic valve replacement. When deemed of high surgical risk, it may be considered the benefit of off-label transcatheter-based therapy [7,11,12], but experience with TAVI for pure AR is limited and has been considered a contraindication for this procedure. The aortic anatomy in native AR patients is often challenging, because they lack annular or leaflet calcification, with the associated risk of insufficient anchoring and valve dislocation [7]. On the other hand, AR often coexists with dilated aortic root or ascending aorta. For this reason, multimodality imaging during pre-procedure planning is of major relevance to accurately measure aortic annulus dimensions and characterize the aortic root morphology. Self-expandable valves offer high and permanent radial forces and have been thought, therefore, to be better suited for treating AR. Roy et al. used a self-expandable CoreValve prosthesis (Medtronic, Minneapolis, MN) in a total of 43 patients with AR. In their series, final implantation was performed in 42 patients. Valve Academic Research Consortium-defined procedure success for TAVI of 74.4% and 18.6% required a second valve due to residual AR. Residual AR≥2+ was reported in 21% [13].

Testa et al. reported a registry of 26 patients treated with CoreValve, with a procedural success of 79%, need for a second valve in 19.2% and residual AR≥2+ in 23% [14].

Seiffert et al. evaluated a second-generation self-expandable device (JenaValve, Munich, Germany) in 31 patients, reporting successful implantation in all cases without any major adverse events. The JenaValve is now the only TAVI device with extended CE mark approval for the treatment of high-risk or inoperable patients with severe AR [15].

Wendt et al. evaluated the transapical ACURATE TA device in eight patients, because it offered tactile feedback and came with the feature of self-positioning at a supra-annular level. The procedure was successful in all patients, without complications and post-procedure AR grade I+ or lower was present in all eight patients [8].

In this case, we decided for the Symetis ACURATE TA device in our patient because of a persisting dissection in the descending aorta that prohibited a transfemoral approach, the existing evidence of the safe anchoring of this device in noncalcified aortic annuli and the successful previous experience in treating another patient with the same valve system.

## Conclusions

This case of a high surgical risk patient shows the feasibility of combined percutaneous intervention for mitral and aortic valve disease, even in patients with off-label indications for transcatheter-based therapy, such as those with severe pure AR. The experience in this case is still scarce and limited to inoperable patients, but as shown recently, patients with off-label indications who were excluded from the trials may have a benefit from the percutaneous approach. Randomized trials and longer follow-up periods are needed to establish a definitive role of TAVI in AR.

## References

[1] Whitlow PL, Feldman T, Pedersen WR (2012). Acute and 12-month results with catheter-based mitral valve leaflet repair: the EVEREST II (Endovascular Valve Edge-to-Edge Repair) High Risk Study. J Am Coll Cardiol.

[2] Kodali SK, Williams MR, Smith CR (2012). Two-year outcomes after transcatheter or surgical aortic-valve replacement. N Engl J Med.

[3] Kische S, D’Ancona G, Paranskaya L (2013). Staged total percutaneous treatment of aortic valve pathology and mitral regurgitation: institutional experience. Catheter Cardiovasc Interv.

[4] Puls M, Seipelt R, Schillinger W (2013). Complete interventional heart repair of multiple concomitant cardiac pathologies in a staged approach. Catheter Cardiovasc Interv.

[5] Madder RD, Safian RD, Gallagher M, Senter SR, Hanzel GS (2011). The first report of transcatheter aortic valve implantation and percutaneous mitral valve repair in the same patient. JACC Cardiovasc Interv.

[6] Barbanti M, Ussia GP, Tamburino C (2011). Percutaneous treatment of aortic stenosis and mitral regurgitation in the same patient: first human cases description. Catheter Cardiovasc Interv.

[7] Krumsdorf U, Haass M, Pirot M, Chorianopoulos E, Katus HA, Bekeredjian R (2011). Technical challenge of transfemoral aortic valve implantation in a patient with severe aortic regurgitation. Circ Cardiovasc Interv.

[8] Wendt D, Kahlert P, Pasa S (2014). Transapical transcatheter aortic valve for severe aortic regurgitation: expanding the limits. JACC Cardiovasc Interv.

[9] Gentles TL, Finucane AK, Remenyi B, Kerr AR, Wilson NJ (2015). Ventricular function before and after surgery for isolated and combined regurgitation in the young. Ann Thorac Surg.

[10] Reardon MJ, Van Mieghem NM, Popma JJ (2017). Surgical or transcatheter aortic-valve replacement in intermediate-risk patients. N Engl J Med.

[11] Ducrocq G, Himbert D, Hvass U, Vahanian A (2010). Compassionate aortic valve implantation for severe aortic regurgitation. J Thorac Cardiovasc Surg.

[12] Dhillon PS, Kakouros N, Brecker SJ (2010). Transcatheter aortic valve replacement for symptomatic severe aortic valve regurgitation. Heart.

[13] Roy D, Laborde JC, Brecker SJ (2013). Transcatheter aortic valve implantation for pure severe native aortic valve regurgitation. J Am Coll Cardiol.

[14] Testa L, Latib A, Rossi ML (2014). CoreValve implantation for severe aortic regurgitation: a multicentre registry. EuroIntervention.

[15] Seiffert M, Bader R, Kappert U (2014). Initial German experience with transapical implantation of a second-generation transcatheter heart valve for the treatment of aortic regurgitation. JACC Cardiovasc Interv.

